# Anxiety, Depression, and Sleep Disorders After COVID-19 Infection

**DOI:** 10.7759/cureus.42637

**Published:** 2023-07-29

**Authors:** Sehnaz Olgun Yıldızeli, Derya Kocakaya, Yüsra Hafza Saylan, Gözde Tastekin, Sudenur Yıldız, Şükran Akbal, Sümeyra Özkan, Hüseyin Arıkan, Sait Karakurt

**Affiliations:** 1 Pulmonary and Critical Care Medicine, Marmara University School of Medicine, Istanbul, TUR

**Keywords:** hospitalization in covid-19, covid anxiety, depression, sleep problems, covid-19

## Abstract

Introduction

As of December 2019, the COVID-19 infection had spread rapidly across the globe, causing a pandemic. Although the virus primarily affects the respiratory and circulatory systems, neuropsychiatric disorders have been reported in a significant number of infected individuals. The aim of this study is to identify anxiety, depression, and sleep disturbances in the early post-COVID period, as well as potential risk factors.

Method

Symptomatic cases whose COVID-19 diagnosis was confirmed by polymerase chain reaction (PCR) positivity within the previous three months were evaluated in the COVID-19 follow-up clinic, where they were observed for at least four weeks after the diagnosis. Cases with no suspicious symptoms and no documented PCR positivity were selected as the control group. All participants completed the Hospital Anxiety Depression Scale (HADS) questionnaire and the Pittsburgh sleep quality questionnaire. The laboratory parameters of hospitalized patients with infection were recorded.

Results

A total of 283 patients were included in the study. While the median age of 144 patients with COVID-19 infection was 44 years, and 104 of them (72.2%) were female, the median age of the controls without COVID-19 infection was 52 years, and 65 of them (46.8%) were female. About 89 (61.8%) of the 144 patients with COVID-19 infections were hospitalized. When the results of the applied HADS questionnaire were analyzed, the median total value of all study participants was 10 points, whereas it was 13 in cases with COVID-19 and nine in those who did not have it (p<0.001). Taking into account the subgroups of the anxiety and depression questionnaires, both results are statistically significantly higher (p<0.001 and p=0.022, respectively) in post-COVID patients. When the hospitalization status of COVID-19 patients was compared, there was no difference in the development of anxiety (p=0.23), but depression(p<0.024) and poor sleep quality(p<0.001) were prevalent in hospitalized patients. The median PSQI score of the entire study population was five points, while it was seven points in cases with COVID-19 infection and four points in cases who did not* *have it (p<0.001). Sleep latency (p<0.003), sleep disturbances (p<0.001), and daytime dysfunction (p<0.001) were statistically significantly worse in COVID-19-infected patients. Female gender (p<0.01) and the presence of past anxiety-depression symptoms (p<0.013) were found to be as risk factors in patients with infection. The correlation between the total HADS score, the PSQI, and the results of the complete blood count and biochemical analysis at the time of diagnosis in hospitalized patients was also investigated. CRP (CI 0.26-0.58) p<0.001 vs (CI 0.09-0.45) p=0.004 and ferritin (CI 0.05-0.43) p=0.017 vs (CI 0.01-0.40) p=0.047exhibited a positive correlation. Similarly, lymphocyte count (CI −0.65 to −0.37) p<0.001 vs (CI −0.39 to −0.01) p<0.001 and lymphocyte percentage (−0.57 to −0.24) p=0.001 vs (−0.65 to −0.37) p=0.039 were negatively correlated.

Conclusion

Early post-infection anxiety, depression, and sleep disturbances increased significantly in COVID-19 patients. Female gender and previous symptoms of anxiety and depression are risk factors, and inpatient treatment increases depression and poor sleep quality. High HADS and poor sleep quality scores are positively correlated with inflammatory parameters and should be evaluated in post-infection in particular.

## Introduction

It has been previously stated previously that certain viral infections of the respiratory tract can cause psychopathological issues during the acute and chronic phases of the disease in some patients [1]. Coronaviruses are a virus family with a single-stranded structure and a viral envelope. Although it is commonly known as the causative agent of the common cold, it has also been identified as the cause of severe acute respiratory syndrome (SARS) [2]. After the severe acute respiratory syndrome (SARS) and Middle East respiratory syndrome (MERS) outbreaks, a variety of neuropsychiatric diagnoses, including post-traumatic stress syndrome, panic attack, depression, and obsessive-compulsive disorder were reported in the short and long-term [3,4].

In March 2020, the World Health Organization (WHO) acknowledged the coronavirus disease (COVID-19), which emerged in the Chinese province of Wuhan in December 2019 and quickly spread around the globe, as a pandemic risk. As the number of cases and experiences with disease affecting all age groups increases, it has been reported that, like other respiratory tract viruses, it causes psychiatric disorders [5]. In cases of infection; delirium, anxiety, depression, and insomnia are among the most commonly reported disorders [6], and large prospective cohort studies have demonstrated that physiological and neuropsychiatric symptoms persist, particularly after hospital discharge or active infection [7]. At the forefront of these findings are studies demonstrating that the infectious effects of coronaviruses originate from physiopathological sequelae or an immune response that directly affects the central nervous system [8]. Coronavirus infection has been shown to cause neural damage in clinical, post-mortem, cell culture, and in vitro studies [9], and it has been reported that neural inflammation, particularly in individuals who have experienced a cytokine storm, plays a significant role in the development of psychiatric symptoms and signs [10].

In addition to the immune and physiological mechanisms, the uncertainty and fear environment brought on by the pandemic, the fear and emotional traumas that develop in those who have a severe illness, and social isolation are considered as environmental factors that contribute to the development of psychiatric symptoms in patients [11]. It has been reported that the incidence of COVID-19 is greater than that of influenza and other respiratory viral pathogens, concerning all these mental problems that occur after viral respiratory infections [12]. In addition to psychiatric symptoms and findings, it was discovered that certain patients also experienced significant insomnia or sleep disturbances, as well as diminished cognitive functions [13]. There are studies indicating that fatigue, insomnia, depression, and shortness of breath are more prevalent during the recovery period of hospitalized patients [14].

The purpose of this study is to determine the prevalence of anxiety and depression in COVID-19 patients, as well as to assess sleep quality and determine its relationship with inflammatory markers.

## Materials and methods

Study design and patient selection

Our study is planned as a cross-sectional observational study. Between October 2021 and March 2022, symptomatic cases with a previous diagnosis of COVID-19 infection documented with polymerase chain reaction (PCR) positivity within the previous three months were evaluated in the COVID-19 follow-up clinic, where they were observed in our hospital for at least four weeks after the diagnosis of active infection. Questionnaires were directed in line with the written informed consent of patients. Patients under the age of 18, pregnant women, and illiterate individuals were excluded from the study. The control group consisted of individuals with no documented COVID-19 infection or clinically suggestive symptoms, and the same questionnaires were administered.

This study was approved by the ethics committee of Marmara University School of Medicine with the approval number 09.2022.167.

Demographic data, clinical parameters, and questionnaires 

Age, gender, marital status, body mass index (BMI), tobacco and alcohol use, comorbid diseases, COVID-19 vaccination status of all patients, and hospitalization status in the study group participating in the study were recorded. The laboratory data (complete blood count, biochemistry, and ferritin values) at the time of diagnosis of hospitalized patients were recorded. Comorbid conditions were evaluated using the Charlson Comorbidity Index (CCI). Cases involving the use of five or more medications were classified as polypharmacy. The diagnosis of a sleep or emotional mood disorder or the presence of associated clinical symptoms prior to the pandemic was also questioned.

A Turkish-validated version of the Hospital Anxiety Depression Scale (HADS) [15], which consists of 14 questions, was used for psychometric evaluation [16]. In the questionnaire, seven questions assessed depression, and seven questions assessed anxiety, with scores ranging from 0 to 21 for each subheading. The Turkish version of the scale has a cut-off of 10 points for anxiety and seven points for depression. Patients with scores exceeding these thresholds were classified as high-risk.

The Turkish-validated version [17] of the Pittsburgh Sleep Quality Index (PSQI) was employed to assess sleep quality [18]. In the questionnaire, which consists of a total of 19 questions and allows us to evaluate sleep quality, quantity, presence, and severity of sleep disturbance, there are seven subheadings that evaluate subjective sleep quality, sleep delay, sleep duration and efficiency, sleeping pill usage, and deterioration in daytime work, with answers ranging from 0 to 3 points. Accordingly, it was given a score of zero if it had never occurred, one if it occurred less than once per week, two if it occurred once or twice per week, and three if it occurred three or more times per week. The sleep quality assessment in the questionnaire was scored as follows: very good=0, fairly good=1, fairly bad=2, and very bad=3 points. The total score ranged from 0 to 21, with high values indicating poor sleep quality and high levels of sleep disorders. Cases with a total score of 5 or higher were classified as having significantly poor sleep quality.

Statistical analysis

The statistical analysis was conducted with the Jamovi 2.3.21 program (The Jamovi Project 2022, Sydney, Australia). Continuous data were expressed as the median and interquartile range (IQR); categorical data were expressed as counts and percentages. The assumption of normality was evaluated using the Shapiro-Wilk test. The Chi-square test is used to compare categorical variables, while the Mann-Whitney U Test is used to compare two independent groups in numerical variables. The Spearman correlation test is used to evaluate variables. If the p-value was less than 0.05, it was considered statistically significant.

## Results

A total of 283 patients were included in the study. While the median age of 144 patients with COVID-19 infection was 44 years and 104 of them (72.2%) were female, the median age of the controls without COVID-19 infection was 52 years and 65 of them (46.8%) were female. About 89 (61.8%) of the 144 patients with COVID-19 infection were hospitalized. Comparing the two groups reveals that CCI, polypharmacy, and vaccination status are comparable. Again, the presence of a sleep disorder or emotional state disorder in the past did not differ between groups. In terms of smoking status, the number of active smokers was higher among those who did not have COVID-19 infection, 41 (29.5%) vs 21 (14.6%), but the number of former smokers was higher among those who did have COVID-19 infection, 14 (9.7%) vs 3 (2.1%) (p<0.001) (Table 1). 

**Table 1:** Demographic properties of the study population. IQR: interquartile range, BMI: body mass index, CCI: Charlson comorbidity index.

	Total (n:283)	COVID-19 (+) (n:144)	COVID- 19 (–) (n:139)	p-value
Age, median (IQR)	48 (26.8)	43 (25)	52 (25)	<0.001
Female, n (%)	169 (59.7)	104 (72.2)	65 (46.8)	<0.001
BMI, median (IQR)	26.5 (6.3)	26 (7.3)	26.7 (5.9)	0.845
Marital status, n (%)				
Married	172 (60.8)	102 (70.8)	70 (50.4)	<0.001
Single	84 (29.7)	36 (25)	48 (34.5)	
Divorced	22 (7.8)	6 (4.2)	16 (11.5)	
Widowed	5 (1.8)	0 (0)	5 (3.6)	
Education status, n (%)				
Primary school graduate	79 (27.9)	67 (46.6)	12 (8.6)	<0.001
High school graduate	87 (30.7)	30 (20.8)	57 (41.1)	
Graduated from university	117 (41.3)	47 (32.6)	70 (50.3)	
Cigarette smoking, n (%)				
Never smoker	206 (73)	108 (75)	98 (70.5)	<0.001
Active smoker	62 (22)	21 (14.6)	41 (29.5)	
Former smoker	17 (5.6)	14 (9.7)	3 (2.1)	
Alcohol consumption, n (%)	116 (40.9)	51 (35,4)	65 (46.8)	0.168
CCI, median (IQR)	1 (2)	1 (2)	1 (2)	0.74
Polypharmacy, n (%)	49 (17.4)	20 (13.9)	29 (20.9)	0.176
COVID-19 vaccination status, n (%)	240 (84.8)	128 (88.9)	112 (80.6)	0.051
Sleep disorder history, n (%)	75 (26.5)	41 (28.5)	34 (24.7)	0.183
Emotional state disorder, n (%)	32 (11.3)	19 (13.2)	13 (9.4)	0.197

When the results of the applied HADS questionnaire were analyzed, the median total value of all study participants was 10 points, whereas it was 13 in cases with COVID-19 and nine in those who did not have it (p<0.001). Taking into account the subgroups of the anxiety and depression questionnaire, the total anxiety score of the group is five, while it is seven in cases with COVID-19 and five in cases who did not. Similarly, the depression score for cases with COVID-19 is six points, compared to five points for cases without the virus, and both results are statistically significant (p<0.001 and p=0.022, respectively) (Table 2). According to this evaluation, 67 (46.5%) cases with COVID-19 infection and 21 (15.1%) cases (p<0.001) in those who did not were detected in the anxiety subgroup. Likewise, 52 (36.1%) vs 21 (15.1%) cases (p<0.01) were detected in the depression subgroup (Table 3).

**Table 2:** HADS score distribution by diagnosis of COVID-19 infection. *All values are represented as median (IQR). HADS: Hospital Anxiety Depression Scale, HADS-A: Hospital Anxiety Depression Scale-Anxiety, HADS-D: Hospital Anxiety Depression Scale-Depression, IQR: interquartile range.

	Total (n:283)	COVID-19(+) (n:144)	COVID-19 (–) (n:139)	p-value
Total HADS score*	10 (9)	13 (13)	9 (6)	<0.001
HADS-A*	5 (4)	7 (7)	5 (3)	<0.001
HADS-D*	5 (5)	6 (7)	5 (3)	0.022

**Table 3:** Frequency of anxiety and depression by diagnosis of COVID-19 infection.

	Total (n:283)	COVID-19 (+) (n:144)	COVID-19 (–) (n:139)	p-value
Anxiety, n (%)	88 (31.2)	67 (46.5)	21 (15.1)	<0.001
Depression, n (%)	73 (25.9)	52 (36.1)	21 (15.1)	<0.01

When the hospitalization status of COVID-19 patients was compared, there was no difference in the development of anxiety (p=0.23), but depression (p<0.024) and poor sleep quality (p<0.001) were prevalent in hospitalized patients.

The median PSQI score of the entire study population was five points, while it was seven points in cases with COVID-19 infection and four points in cases who did not (p<0.001). While subjective sleep quality did not differ between the groups, sleep latency (p<0.003), sleep disturbances (p<0.001), and daytime dysfunction (p<0.001) were statistically significantly worse in COVID-19-infected patients. While sleep duration (p<0.005) and habitual sleep efficiency (p<0.001) were found to be significantly better compared to those without COVID-19 infection (Table 4). When sleep quality was evaluated according to the general evaluation of the test, 106 (73.6%) patients who had COVID-19 infection had poor sleep quality, compared to 56 (40.3%) patients who did not have COVID-19 (p<0.001) (Table 5).

**Table 4:** PSQI by diagnosis of COVID-19 infection. *All values are represented as median (IQR). PSQI: Pittsburgh Sleep Quality Index, IQR: interquartile range.

	Total (n:283)	COVID-19 (+) (n:144)	COVID-19 (–) (n:139)	p-value
PSQI total*	5 (4)	7 (4)	4 (3)	<0.001
Subjective sleep quality*	1 (1)	1 (1)	1 (0)	0.182
Sleep latency*	1 (1)	2 (1)	1 (1)	0.003
Sleep duration*	1 (1)	0 (1)	1 (0)	0.005
Habitual sleep efficiency*	1 (1)	0 (1)	1 (1)	<0.001
Sleep disturbances*	1 (2)	2 (1)	0 (1)	<0.001
Use of sleep medication*	0 (0)	0 (0)	0 (0)	0.011
Daytime dysfunction*	0 (1)	1 (2)	0 (0)	<0.001

**Table 5:** Sleep quality by diagnosis of COVID-19 infection.

	Total (n: 283)	COVID-19 (+) (n: 144)	COVID-19 (–) (n: 139)	p-value
Good sleep quality, n (%)	118 (42.1)	35 (24.3)	83 (59.7)	<0.001
Bad sleep quality, n (%)	162 (57.9)	106 (73.6)	56 (40.3)	

When the patients with the diagnosis of COVID-19 were divided into inpatient and outpatient treatment groups, there was no difference in the frequency of anxiety (p=0.21), but poor sleep quality as measured by PSQI scores (p<0.001) and depression (p<0.024) were more prevalent in inpatients.

Age (p=0.49), CCI (p=0.27), pack-years of smoking (p=0.82), polypharmacy (p=0.09), and past sleep disorder complaints (p=0.08) were not found to be risk factors for the development of anxiety and depression in infected cases. However, female gender (p<0.01) and the presence of past anxiety-depression symptoms (p<0.013) were found to be risk factors. Age (p=0.053) remained within the statistical significance limit for depression, while female gender (p<0.15), CCI (p=0.17), pack-years of smoking (p=0.96), polypharmacy (p=0.43), previous sleep disorder complaints (p=0.25), and previous anxiety-depression symptoms (p<0.97) did not differ.

**Figure 1:**
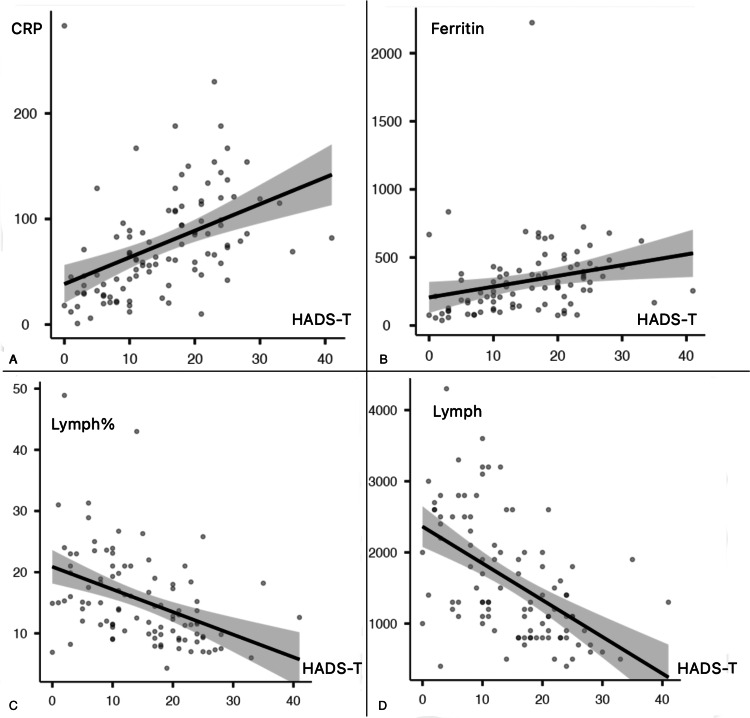
Correlations between HADS-T and inflammatory markers. HADS-T: Hospital Anxiety and Depression Scale Total Score, CRP: C-reactive protein, Lymph%: lymphocyte percent on complete blood count, Lymph: lymphocyte count on complete blood count.

**Figure 2:**
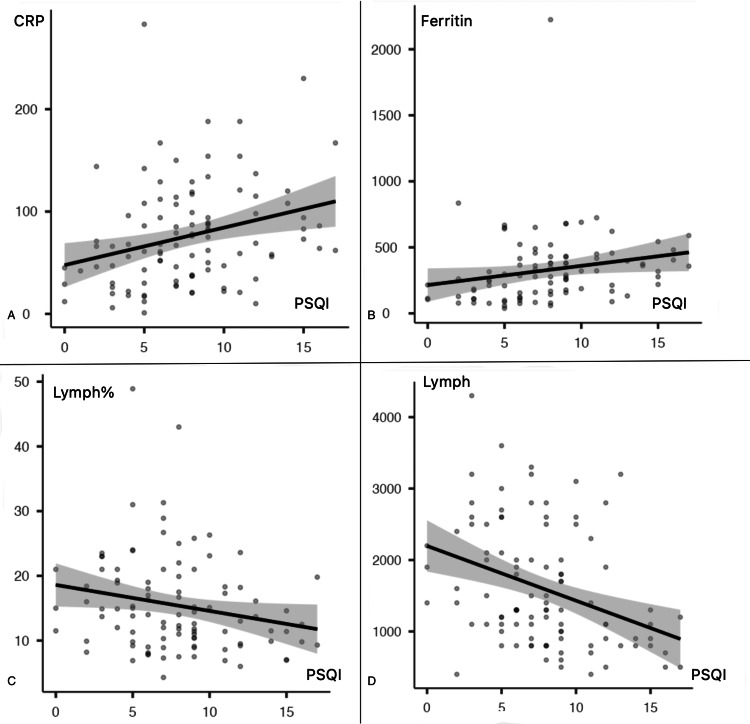
Correlations between PSQI and inflammatory markers. PSQI: Pittsburgh Sleep Quality Index, CRP: C-reactive protein, Lymph%: lymphocyte percent on complete blood count, Lymph: lymphocyte count on complete blood count.

A strong positive correlation (p<0.001) was discovered between the HADS total score and PSQI scores of all populations included in the study. The correlation between the total HADS sore, the PSQI, and the results of the complete blood count and biochemical analysis at the time of diagnosis in hospitalized patients was also investigated. CRP (CI 0.26-0.58) p<0.001 vs (CI 0.09-0.45) p=0.004 and ferritin (CI 0.05-0.43) p=0.017 vs (CI 0.01-0.40) p=0.047 exhibited a positive correlation. Similarly, lymphocyte count (CI −0.65 to −0.37) p<0.001 vs (CI −0.39 to −0.01) p<0.001 and lymphocyte percentage (−0.57 to −0.24) p=0.001 vs (−0.65 to −0.37) p=0.039 were negatively correlated (Figures 1, 2) (Table 6).

**Table 6:** Correlation between laboratory parameters of patients with COVID-19 infection and HADS score and PSQI. r: correlation coefficient, CI: 95% confidence interval, HADS: Hospital Anxiety Depression Scale, PSQI: Pittsburgh Sleep Questionnaire Index, CRP: C-reactive protein.

	HADS-T			PSQI		
	r	CI	p-value	r	CI	p-value
HADS-total	N/A			0.557	0.45–0.63	<0.001
PSQI	0.557	0.45–0.63	<0.001	N/A		
CRP	0.433	0.26–0.58	<0.001	0.284	0.09–0.45	0.004
Ferritin	0.251	0.05–0.43	0.017	0.209	0.01–0.40	0.047
Lymphocyte count	–0.527	–0.65 to –0.37	<0.001	–0.35	–0.39 to –0.01	<0.001
Lymphocyte%	–0.422	–0.57 to –0.24	0.001	–0.209	–0.65 to –0.37	0.039

## Discussion

We evaluated the anxiety, depression, and sleep quality of cases with a diagnosis of COVID-19 infection within the previous three months. The scores of those with the infection were significantly higher, and their anxiety was more pronounced than that of those who did not have the infection. Again, in the sleep quality evaluation, the scores of the patients with the infection were high, and the sleep quality was rated as bad, and this significance was maintained in the subgroup analysis. CRP and ferritin values, which are also known as determinants of COVID severity, were positively correlated with high scores, whereas lymphocyte count and percentage had a negative correlation.

Pathological psychiatric findings detected after COVID-19 infection can be exacerbated by the fear of death or serious illness, the fear of transmitting the disease or infecting family members, and the contribution of social isolation, as well as the inflammatory and immune response caused by the virus. It has been demonstrated that patients with prolonged COVID or post-COVID syndrome complain of weakness, fatigue, depression, sleep disturbance, concentration, and memory difficulties [19,20]. Although the cause of the prolonged symptoms is unclear, it is believed that they are related to the inflammation in the foreground to blame. Viruses that remain in the lungs during the recovery period, especially in cases of severe infection, have been linked to persistent symptoms resulting from chronic inflammation, activated complement system, and emerge of micro- and macro-thrombi [21,22]. The pillars of the immune response are the cytokines, chemokines, and other inflammatory mediators that begin locally and become systemic after viral infection. In addition to the previously detected activation of interleukin 1-β (IL-1β), IL-6, tumor necrosis factor (TNF-α), and T-helper-1 in SARS and MERS outbreaks, IL-4, IL-10, and T-helper-2’s were also found to be activated in COVID-19 infection [23-25]. It has been suggested that dysregulation of cytokines, particularly IL-1-β, IL-6, IL-10, interferon (IFN-γ), and TNF-α, contributes to the pathogenesis of certain psychiatric disorders [26,27].

Anxiety and depression have been reported as the most prevalent psychiatric disorders in acute, prolonged, and post-COVID periods [28]. In a meta-analysis, the prevalence of anxiety was 14-44% and the prevalence of depression was 19.2-21.5% in cases with infection in the first four months [29]. However, in other studies evaluating the presence of anxiety and depression in the sixth and 12th months after infection, the prevalence of anxiety and depression was 23% and 26%, respectively [12,30]. According to studies evaluating gender differences in the development of anxiety and depression, this tendency is more prevalent in women [31,32]. In addition, it was emphasized that post-infectious anxiety and depression are more prevalent in patients with underlying psychiatric symptoms or a diagnosis of psychiatric disorders [31]. In our study, anxiety was found to be 46.5% in cases with a positive history of COVID-19, while it was 15.1% in cases without a history of infection, and depression was found as 36.1% in cases with a history of infection and 15.1% in cases without a history of infection. In our study, which encompasses the first three months after infection, the rates and predominance of anxiety are comparable to those reported in the literature. Female gender and the presence of symptoms related to anxiety and depression in the pre-COVID period were found to be risk factors for the development of post-COVID anxiety, similar to previous research. Although there was a correlation between advanced age and post-COVID depression, it did not make statistical significance.

In cases of severe infection and hospitalization, cognitive and mental impairments, as well as anxiety, depression, and poor sleep quality, were reported in studies. Nakanishi et al. examined patients who were followed up in the intensive care unit and reported post-intensive care syndrome (physical, cognitive, and mental deterioration) between 50% and 70% in non-COVID-19 infections, whereas they reported physical impairment between 28% and 87%, cognitive impairment between 20% and 57%, and mental health problems between 6% and 60%, mainly anxiety and depression, after the first six-month period of COVID-19 infection [33].

In the study conducted by Vlake et al., which evaluated the post-discharge anxiety, depression, and post-traumatic stress symptoms of hospitalized COVID-19-infected patients, it was found that 25% of the patients experienced psychological distress [34]. In another study of COVID-19 survivors, patients with a history of severe illness were found to have a prevalence of 29% for sleep disorders, and anxiety and depression were found to be more common in young patients, especially if there was a previous depressive symptom [35]. Since hospitalizations and intensive care stays of the patients in our study occurred in different centers, the indications for hospitalization and the intensity of intensive care varied. The length of hospitalization and intensive care admissions were therefore not taken into account. However, the inflammatory parameters of the hospitalized patients correlated positively with anxiety and depression scores. Again, supporting this, a negative correlation was found between the lymphocyte count and percentages and these scores. High inflammatory parameters and low lymphocyte counts support the severe illness, and similar results were obtained in other studies [1,2,23]. Outpatient or inpatient treatment for COVID-19 did not differ in terms of anxiety, but hospitalization was associated with an increased risk for the development of depression and poor sleep quality.

Considering the limitations of the study, the history of infection and hospitalization was questioned retrospectively, and there are insufficient data regarding this. Due to the variance in time between the onset of the first symptom and the time of hospital admission, bias may have been introduced by using the laboratory results from the initial hospital admission. Due to the lack of clear data on the length of stay and intensive care requirements of patients receiving inpatient treatment at various centers, these data cannot be included in the study. Again, a severity-based evaluation was not possible due to the lack of sufficient data on patients receiving inpatient treatment. In our study, anxiety, depression, and sleep disorders were evaluated, but cognitive dysfunction, which is assumed to be common in these patients, was not possible to assess. Last but not least, patients who describe sleep and psychiatric disorder symptoms in advance do not have a medical evaluation of this, but rather subjective data.

## Conclusions

In conclusion, anxiety, depression, and poor sleep quality were found to be more prevalent in early post-COVID patients compared to those without infection. The female gender and the presence of symptoms related to anxiety and depression prior to infection were identified as risk factors for anxiety. High levels of inflammatory parameters indicating severe disease are positively correlated with anxiety, depression, and poor sleep quality. Again, depression and poor sleep quality increased significantly in hospitalized patients. Inquiring about this during the outpatient follow-up of these patients will improve their quality of life, and compliance with treatment and accelerate the transition to total well-being.
